# ChatGPT: revolutionizing cardiothoracic surgery research through artificial intelligence

**DOI:** 10.1093/icvts/ivad090

**Published:** 2023-06-22

**Authors:** Arian Arjomandi Rad, Peyman Sardari Nia, Thanos Athanasiou

**Affiliations:** Medical Sciences Division, University of Oxford, Oxford, UK; Department of Cardiothoracic Surgery, Maastricht University Medical Center, Maastricht, Netherlands; Department of Surgery and Cancer, Imperial College London, London, UK; Department of Cardiothoracic Surgery, Maastricht University Medical Center, Maastricht, Netherlands; Department of Surgery and Cancer, Imperial College London, London, UK

**Keywords:** ChatGPT, Cardiothoracic surgery, Research, Artificial intelligence

## BACKGROUND

**Figure 1:**
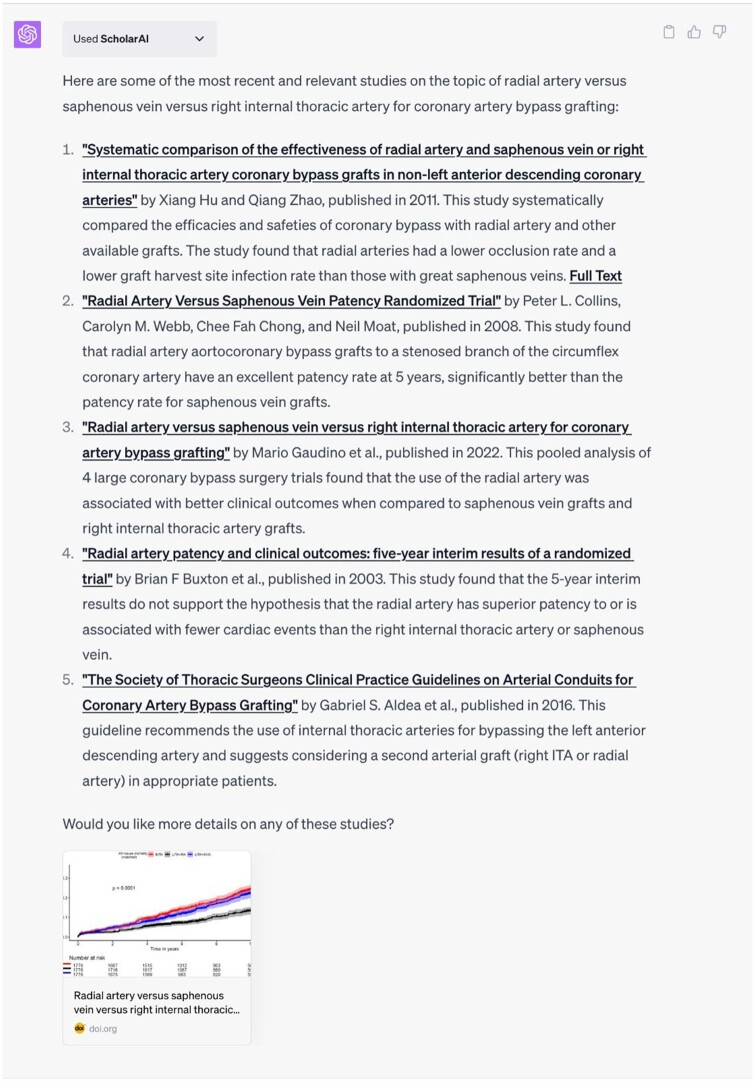
Response from ChatGPT [4] when asked to provide the most recent and relevant references in saphenous vein versus radial artery versus right internal thoracic artery for coronary artery bypass grafting (CABG).

The integration of artificial intelligence (AI) and natural language processing (NLP) into medical practice is rapidly accelerating, opening new avenues for diagnosing and treating diseases and advancing personalized healthcare [1–3] (Fig. 1). The potential of these technologies is particularly significant in the sphere of cardiothoracic surgery research.

Cardiothoracic surgery is a dynamic and complex field, characterized by constant evolution in techniques, technologies, and patient outcomes. As a result, physicians and researchers are constantly looking for novel approaches to improve patient care. In this pursuit of continuous improvement, AI and NLP are proving to be effective tools, especially when used for large-scale data analysis.

One benefit of incorporating AI and NLP into cardiothoracic surgery research is the ability to identify significant patterns in huge datasets. With the introduction of electronic medical records and other digital health data repositories, a vast data environment for analysis has been established. However, this volume of data comes with its own difficulties, making it problematic for researchers and physicians to extract insights that may be put to use. Here, machine learning methods are used by AI and NLP to interpret large datasets and find patterns and trends that might escape manual study.

Additionally, NLP has the singular capacity to interpret and analyse unstructured data, such as free-text clinical notes. This makes it possible to glean priceless information about patient outcomes and disease trajectories, providing a richer, more nuanced picture of the changing cardiothoracic surgery landscape.

This editorial seeks to explore how ChatGPT is catalysing the evolution of cardiothoracic surgery research, shaping the future of this dynamic speciality.

## ChatGPT IN CARDIOTHORACIC SURGERY RESEARCH: THE BASICS

One of the most promising AI models currently available is ChatGPT (California, USA) [4], a language model developed by OpenAI. By utilizing a strong language model that has been trained on a variety of data sources, such as academic literature and medical textbooks, ChatGPT harnesses the power of machine learning. Its strength is in its ability to examine and comprehend vast amounts of data, providing significant insights that could revolutionize cardiothoracic research and practice. While ChatGPT has mostly been utilized for text production and other NLP tasks, such as language translation, interest in its potential uses in healthcare is growing.

**Figure 2:**
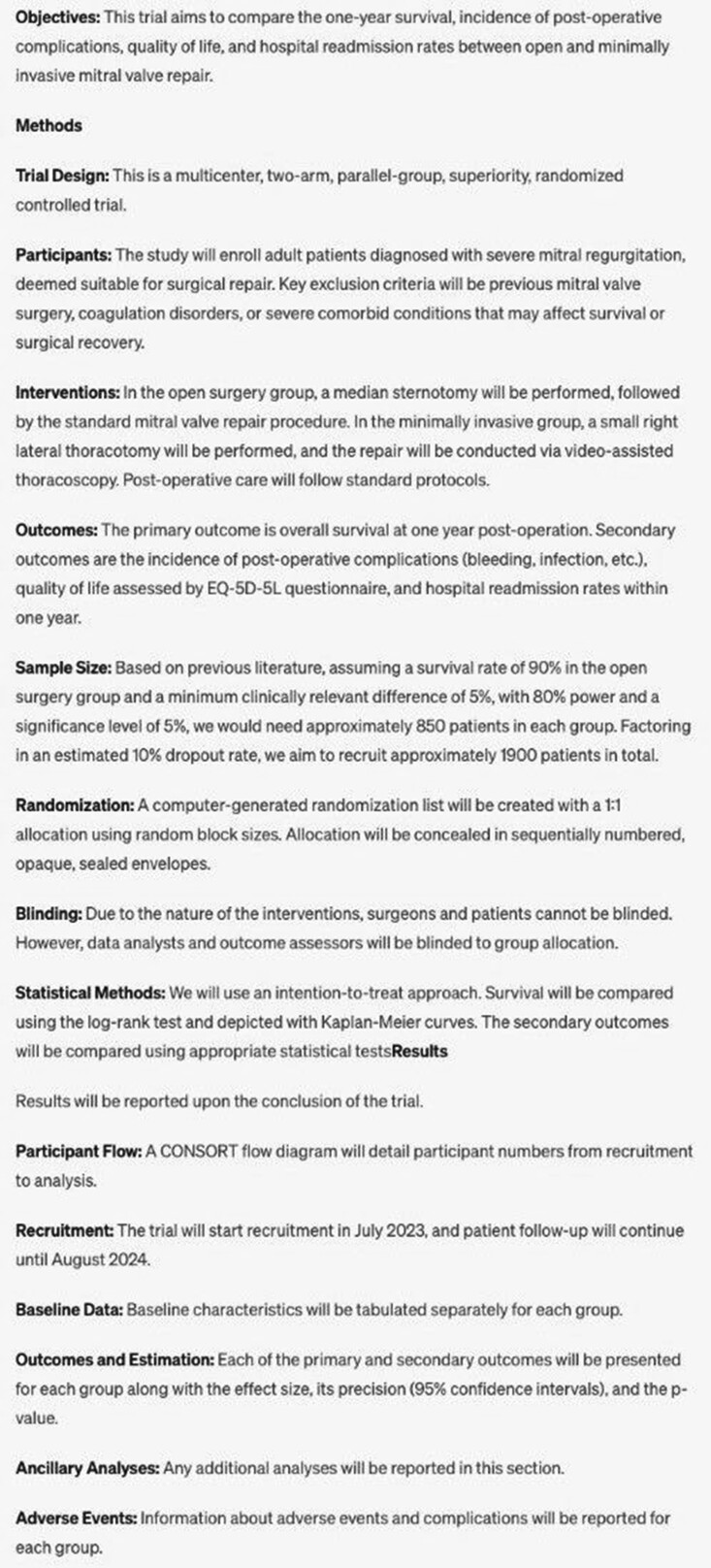
Response from ChatGPT [4] when asked to provide an example of a trial design following the CONSORT statement, focussed on open versus minimally invasive mitral valve surgery.

**Figure 3:**
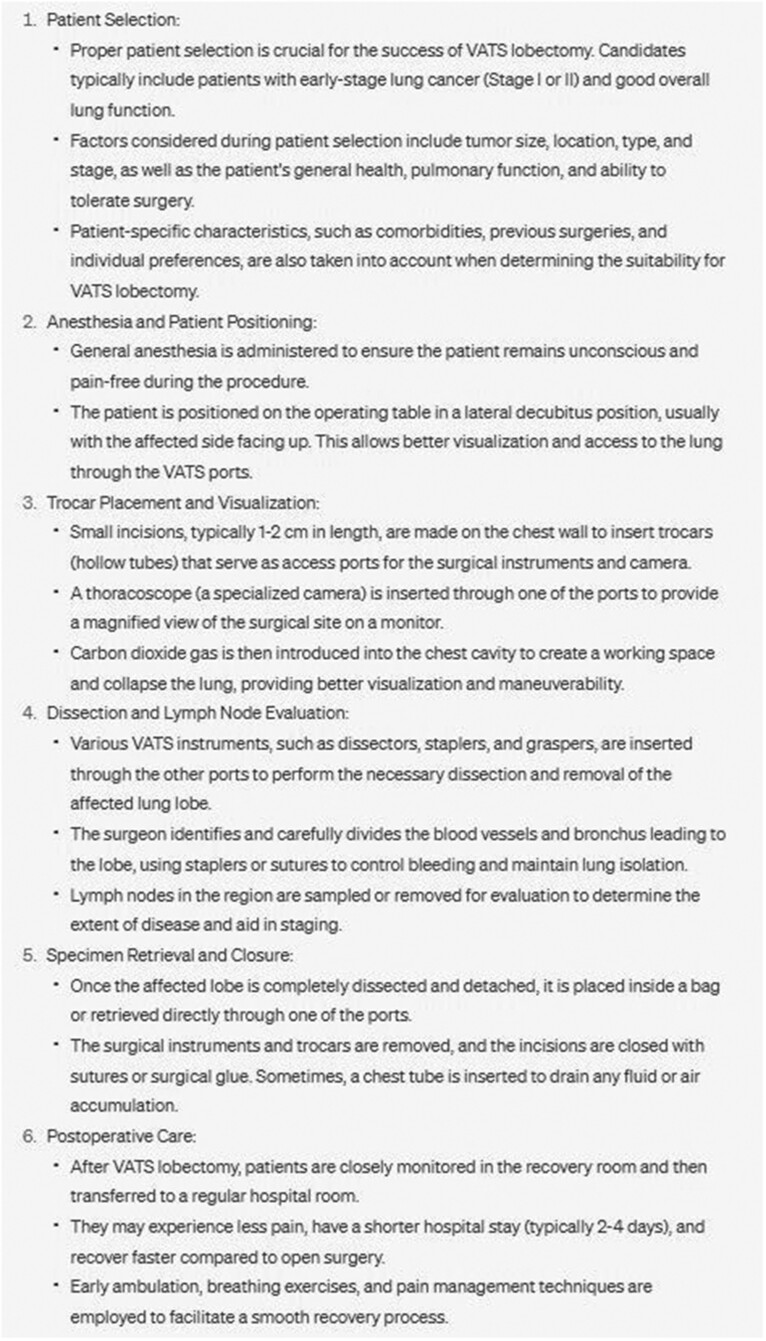
Response from ChatGPT [4] when asked to explain the fundamentals of video-assisted thoracoscopic surgery lobectomy to a medical student.

1. Literature review: Given the breadth of medical literature, ChatGPT could assist in quickly sifting through hundreds of papers to help researchers find the most relevant and recent studies for their own research or to stay up to date with the latest advancements in the field (Fig. 1).
2. Data interpretation and analysis: ChatGPT could provide guidance on interpreting complex datasets, explaining statistical models, and suggesting relevant ones for certain types of research based on a pre-existing database of information.
3. Research design: ChatGPT could offer suggestions on how to design studies, what variables to include, and how to control for confounding factors based on a broad understanding of research methodologies in the field (Fig. 2).
4. Predictive modelling: Based on available public health data, ChatGPT could assist in generating predictive models for surgical outcomes, postoperative care, and patient recovery timelines.
5. Collaboration facilitation: ChatGPT could help researchers collaborate by summarizing and explaining complex concepts, and facilitating communication between professionals from different specialities involved in cardiothoracic surgery research.
6. Writing assistance: Researchers could leverage ChatGPT to draft abstracts, manuscripts, or grant proposals, with the AI providing initial versions that researchers can then refine and edit.
7. Educational resource: ChatGPT could be used to clarify complex topics, provide deeper context, and supply relevant references for researchers, students, and trainees within the field of cardiothoracic surgery (Fig. 3).
8. Hypothesis generation: By integrating and analysing vast amounts of information, ChatGPT may assist in generating novel hypotheses for future research, potentially leading to discoveries and advancements in cardiothoracic surgery.

## ADVANCED USE OF ChatGPT IN THE CONTEXT OF CARDIOTHORACIC SURGERY

- Outcome prediction: ChatGPT may help in the creation of predictive models by utilizing the strength of sizable patient data sets. These models can help clinicians identify individuals who may need more aggressive treatment plans or who have a higher risk of problems. This use of AI might make it easier to make early interventions, which might enhance patient outcomes.
- Identification of new therapeutic targets: ChatGPT may be key in identifying new therapeutic targets for lung and cardiovascular illnesses. ChatGPT could identify new genes or proteins associated with the development or course of a disease by processing vast amounts of genomic and proteomic data. This might spur the creation of fresh treatments that target the identified molecules, thereby improving patient outcomes.
- Refinement of surgical techniques: Furthermore, ChatGPT can contribute to the swift development of advanced surgical techniques. ChatGPT could produce prediction models that direct clinicians in deciding the best surgical strategy for specific patients by analysing substantial patient data and surgical outcomes. This might make it unnecessary to do extensive testing and validation, hastening the creation and application of novel surgical procedures.
- Enhanced patient engagement: ChatGPT could be harnessed to construct conversational agents or chatbots, enabling patients to gain information about their conditions, treatment options, and other healthcare topics. This could empower patients by enhancing their understanding of their health status while providing a platform for questions and support beyond the clinical environment. Moreover, chatbots powered by ChatGPT could gather patient-reported outcomes data, aiding clinicians in understanding patients' status between visits and facilitating informed treatment decisions.
- Guideline adherence: Adherence to clinical guidelines is paramount for delivering high-quality care, yet staying current with evolving recommendations and consistently applying them can be challenging. ChatGPT could contribute to the development of decision support tools that offer clinicians real-time guidance on managing specific conditions based on the latest evidence-based guidelines, thereby enhancing the quality of care and reducing variability in practice.
- Personalized treatment: ChatGPT's potential impact on personalized treatment plans could be significant. Given the highly individualized nature of cardiothoracic surgery, treatment plans often require tailoring to the specific needs of each patient. The development of these personalized plans, however, can be complex and time-consuming, necessitating comprehensive analysis of patient data and medical history. By employing ChatGPT's advanced NLP capabilities, clinicians and researchers could more efficiently extract and analyse unstructured data, such as free-text clinical notes, to create bespoke treatment plans for every patient.

## ChatGPT API IN RESEARCH: ADVANCED USE

The OpenAI GPT API is a powerful tool that allows developers to interface with GPT models like ChatGPT. By utilizing this API, developers can tailor interactions to suit a wide range of applications. In the context of research, here are some ways it could be customized:

1. Fine-tuning with specialized data: ChatGPT can be further fine-tuned using specialized medical literature or surgical procedure manuals, even though it is pre-trained on a sizable dataset. This might make it possible for it to offer responses that are more precise and tailored to the field of cardiothoracic surgery.
2. Interactive learning systems: Including AI capabilities in these systems can improve the quality of medical education. A system might be created, for instance, to mimic various patient scenarios and prompt the user to make clinical decisions. The AI may then give the user feedback on their choices, outlining their justifications and offering changes based on industry best practises.
3. Hybrid models: Combining the capabilities of different AI models could provide more comprehensive solutions. For instance, pairing ChatGPT's NLP with an AI model specifically designed for analysing medical imaging could offer powerful diagnostic and prognostic tools.
4. Predictive analytics: Cardiothoracic surgery could benefit significantly from the development of AI models that can manage massive anonymized datasets and make precise predictions. For instance, based on several variables such as patient age, existing diseases, and type of surgery, these models could forecast patient outcomes. By assisting users in comprehending the statistical methods applied and interpreting the outcomes, ChatGPT might be of assistance.
5. Integrating AI into current systems: Adding AI capabilities to electronic health records or other hospital systems may improve productivity and judgement. For instance, AI could assist clinicians in swiftly identifying pertinent patient data, alerting them to potential hazards, or suggesting individualized treatment strategies based on previous patient records.
6. Improving user interfaces: User interaction with AI can also be enhanced, making it easier for healthcare professionals to use AI tools. By making user interfaces more intuitive, reducing technical jargon, and presenting information clearly and succinctly, AI can become a more effective tool in the medical field.

## USING ChatGPT IN DAILY PRACTICE: A SIMPLIFIED EXAMPLE

1. Literature review:
  - Research team: ‘ChatGPT, can you search for recent articles on mitral valve surgery techniques?’
  - ChatGPT (browser tool and ScholarAI plugin): *Searches for the query and presents a list of recent articles.*
  - Research team: ‘ChatGPT, can you summarize the main points of these three articles?’
  - ChatGPT: *Summarizes the key findings from the articles selected.*
2. Data interpretation and analysis:
  - Research team: ‘ChatGPT, can you explain the statistical model used in this study?’, provide URL to PDF file.
  - ChatGPT (using ChatWithPDF plugin): *Provides a simplified explanation of the statistical model.*
3. Research design:
  - Research team: ‘ChatGPT, based on the current literature, what variables should we consider for our study design?’
  - ChatGPT (use 4.0 version): *Suggests relevant variables and confounding factors.*
4. Predictive modelling:
  - Research team: ‘ChatGPT, can you guide us on how to build a predictive model for postoperative outcomes based on these variables?’
  - ChatGPT: *Guides the team through the process, suggesting appropriate statistical tools and models.*
5. Collaboration facilitation:
  - Research team: ‘ChatGPT, we need to explain our model to the cardiothoracic surgeons. Can you help us simplify it?’
  - ChatGPT: *Provides a simplified and understandable version of the model.*
6. Writing assistance:
  - Research team: ‘ChatGPT, can you help us draft the abstract for our research paper to be submitted to the EACTS annual conference?’
  - ChatGPT: *Generates a draft of the abstract based on the provided information.*
7. Educational resource:
  - Research team: ‘ChatGPT, we have some medical students who need a better understanding of mitral valve surgery. Can you provide a comprehensive explanation?’
  - ChatGPT: *Explains mitral valve surgery, its indications, techniques, and postoperative care.*
8. Hypothesis generation:
  - Research team: ‘ChatGPT, based on the current literature and our data, what are some potential hypotheses for our next research project?’
  - ChatGPT (API or 4.0 version preferred): *Proposes several hypotheses for further research.*

## NOT ALL THAT SHINES IS GOLD: CHALLENGES, LIMITATIONS, ETHICAL AND LEGAL IMPLICATIONS

**Figure 4:**
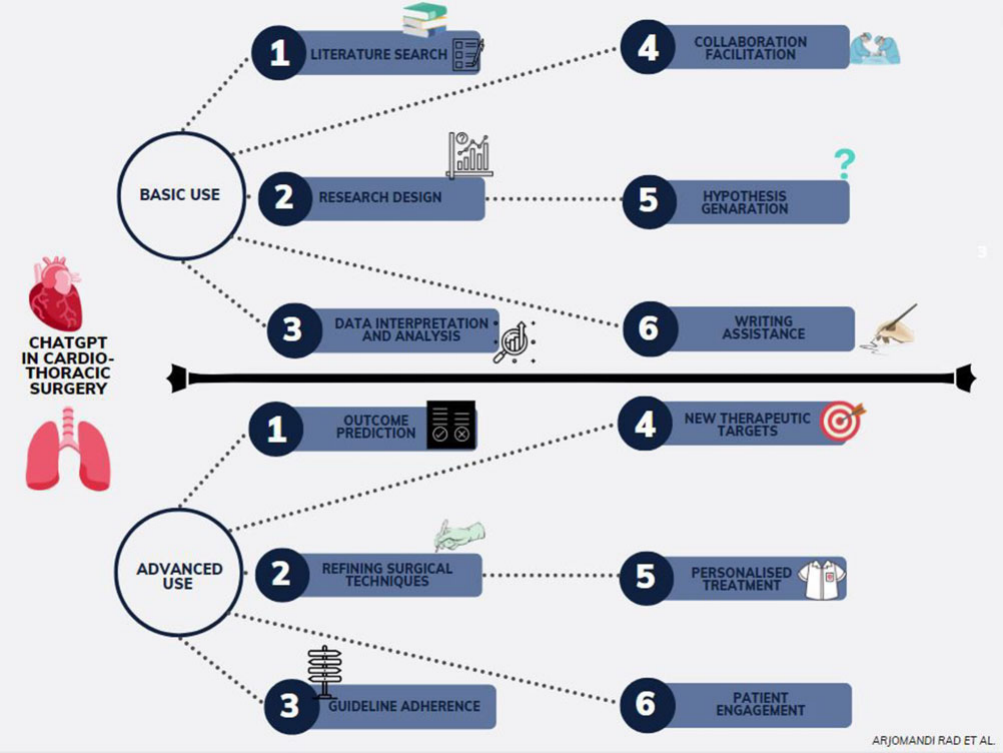
Summary of the main avenues for research in cardiothoracic surgery with ChatGPT.

ChatGPT's application in cardiothoracic surgery research presents promising potential (Fig. 4), yet it comes with significant challenges that require careful attention. The correctness and representativeness of the data used to train ChatGPT are of particular relevance. Predictive models may be unreliable as a result of biased or missing data. A multidisciplinary team of clinicians, data scientists, and AI specialists should work together to prevent this by ensuring data quality and the transparency of the models that are created.

Another issue with AI models like ChatGPT is their opaque decision-making process. Clinicians can be hesitant to use a tool whose operation they are unfamiliar with. As a result, scientists are looking at how to develop AI models that have clear justifications for their decisions.

The ethical and legal implications of using ChatGPT in cardiothoracic surgery research must be taken into account. Despite being an effective tool for data analysis, ChatGPT should not replace the knowledge and discretion of medical experts. Decisions based on ChatGPT analysis should be made in consultation with medical experts and in accordance with clinical best practises and recommendations.

- Patient privacy and confidentiality are paramount. Data used for training or testing must be de-identified, and stringent security measures should be in place to prevent unauthorized access or data breaches.
- Potential bias in the data or model used to train ChatGPT is another concern. The data should be representative of the patient population under study, and any biases should be identified and addressed.
- Legal implications may arise if a patient is harmed due to a decision based on ChatGPT analysis, raising questions about liability. Researchers and clinicians should have appropriate insurance, institutional ethical board approval, and follow established protocols for handling such issues.

Researchers and clinicians should work with ethicists, legal experts, and patient advocates to create specific guidelines for ChatGPT's usage in cardiothoracic surgery research to manage these ethical and legal problems. This includes obtaining fully informed consent from patients, addressing potential biases in data and models, guaranteeing model transparency, and establishing procedures for dealing with moral or legal dilemmas. By doing this, it will be possible to harness the potential of AI to enhance patient outcomes and increase medical knowledge while also making sure that ethical and legal concerns are properly taken into account.

What is more, the development of frameworks is required for the adoption and deployment of ChatGPT or comparable AI language models in general research and cardiothoracic surgery research. These guidelines are essential for assuring the responsible and efficient use of these potent technologies. They give institutions, researchers, and physicians instructions on how to use ChatGPT into their processes. These frameworks support the preservation of patient confidentiality, the reduction of biases, and the potential limitations of AI models by outlining specific standards for data privacy, ethical concerns, validation processes, and transparency. Additionally, frameworks encourage researcher cooperation and knowledge exchange, facilitating group learning and the creation of best practises. They are crucial to realizing the full potential of AI language models while preserving moral principles and advancing rigorous scientific investigation.

## CONCLUSION

AI and NLP stand poised to catalyse a transformative shift in cardiothoracic surgery research, with ChatGPT emerging as a pivotal instrument in this evolution. Despite existing challenges and limitations, the potential advantages of integrating ChatGPT into cardiothoracic surgery research are substantial. It is incumbent upon researchers and clinicians to explore innovative strategies to harness this technology's power for enhancing patient outcomes. Through collaborative efforts and capitalizing on the strengths of AI and NLP, we can propel the field of cardiothoracic surgery forward, thereby improving patient lives globally.

**Conflict of interest:** Peyman Sardari Nia has a consultancy agreement with Neochord Inc, Edwards Lifesciences, Medtronic, Abbott and Fuijfilm Medical and is the inventor of MV simulators that are commercialized through his start-up (Simurghy). The other authors report no conflicts of interest.
